# Stabilization of Vancouver B Periprosthetic Femur Fractures With Cerclage Wiring: A Retrospective Chart Review

**DOI:** 10.7759/cureus.25063

**Published:** 2022-05-17

**Authors:** Ajith Malige, Matthew Beck, Frederick Mun, Maddie Goss, Henry Boateng, Chinenye Nwachuku

**Affiliations:** 1 Orthopaedic Surgery, St. Luke's University Health Network, Bethlehem, USA; 2 Orthopaedic Surgery, Philadelphia College of Osteopathic Medicine, Philadelphia, USA; 3 Orthopaedic Surgery, Penn State University College of Medicine, Milton S. Hershey Medical Center, Hershey, USA

**Keywords:** cerclage, arthroplasty, fracture, femur, periprosthetic

## Abstract

Background: While biomechanical and clinical studies detailing the equivalence and, sometimes, the superiority of cerclage wiring fixation compared to plate fixation in select fractures (Vancouver B1 and C) exist, no studies exist detailing outcomes after cerclage wiring fixation in all Vancouver B fracture types. This study explores whether there is a difference in clinical outcomes between Vancouver B fractures fixed with cerclage wiring and those without.

Methods: This retrospective multicenter study reviewed 295 patients from 2007 to 2018 with periprosthetic femur fractures. Vancouver B periprosthetic fractures stabilized utilizing cerclage wiring were identified and compared against fractures stabilized without cerclage wiring, with 33% being B1, 48.4% B2, and 18.6% B3 fractures. Demographics, injury details, fracture classification, surgical details, fracture union, and postoperative complications were recorded for each patient.

Results: A majority of our patients were females (65.9%) and were older than 71 years of age (65.6%) without diabetes (63.3%) or smoking history (92.2%). Sixty-nine patients progressed to fracture union (76.7%), two (2.2%) to delayed union, and 19 (21.1%) to nonunion. There was no difference in the union rate (p = 0.98) or time to union (p = 0.91) between the fixation methods. Finally, there was no difference in the infection rate (p = 0.81), re-fracture rate (p = 0.87), or re-operation rate (p = 0.75) between the fixation methods.

Conclusion: Periprosthetic femur fractures are common injuries, most commonly occurring after low-energy mechanisms in the elderly female population. While the Vancouver fracture pattern helps to guide the surgical construct used for fixation, the use of cerclage wires does not impact bony union in these injuries. Interestingly, increasing age and female gender were associated with increased union rates. Surgeons should individually consider each patient's demographic as well as fracture type when deciding which construct will achieve stable fixation that allows for fracture healing.

## Introduction

Periprosthetic femur fractures can potentially compromise a patient’s life and lead to a debilitating loss of function, posing a complex challenge to patients and surgeons alike. With the increasing incidence of these fractures secondary to both an aging population as well as the rise in the number of total hip arthroplasty, it is paramount to determine biomechanically and clinically strong fixation methods that stabilize the femur-prosthesis complex while allowing for fracture healing [1,2].

Periprosthetic femur fractures can either occur intra-operatively or postoperatively. Existing literature reports the incidence of fracture after total hip arthroplasties (THA) as high as 6% [3-5], with increased rates found in revision versus primary surgeries [6], uncemented versus cemented prosthetics [7], and revision surgeries with impaction allografting [8]. Stress risers, such as osteolytic lesions, varus malposition of existing implants, and impingement of a loose stem against the lateral cortex can further increase the risk of fractures in these patients [7]. Surgeons must also be aware of all patient demographic factors that increase the risk of periprosthetic femoral fractures, including female gender, tobacco use, immunosuppression, and osteoporosis [3,7,9-11].

These fractures are often the result of low-energy injuries (falls during activities of daily living) [9] and are often associated with prosthetic loosening [11]. The Vancouver classification system [12-14] is most commonly used to classify periprosthetic femur fractures and guide treatment based on the fracture location, the stability of prosthesis, and the quality of surrounding bone. Due to high morbidity [14], high complication rates, and poor functional outcomes associated with this injury, operative intervention is recommended as the first line for most patients [15]. Biomechanical [16] and clinical [17] studies have compared the various fixation methods used to stabilize these fractures. This includes plate fixation, revision arthroplasty, allograft supplementation, and cerclage wiring [18].

While there are a few biomechanical and clinical studies detailing the equivalence [19-21] and sometimes superiority [22] of cerclage wiring fixation compared to plate fixation in select fractures (Vancouver B1 and C), no studies exist detailing clinical outcomes specifically after cerclage wiring fixation in all Vancouver B fracture types. This study specifically explores cerclage wiring fixation after Vancouver B fracture, comparing whether there is a difference in the clinical outcomes between the fractures fixed with cerclage wiring (with or without plate fixation) and those without cerclage wiring (utilizing only plate fixation, prosthesis exchange, or a combination of the two). The authors hypothesize that there is no clinical difference in the outcomes between the fracture fixation methods for periprosthetic femur fractures, and these injuries can be adequately stabilized with or without cerclage wiring.

## Materials and methods

**Figure 1:**
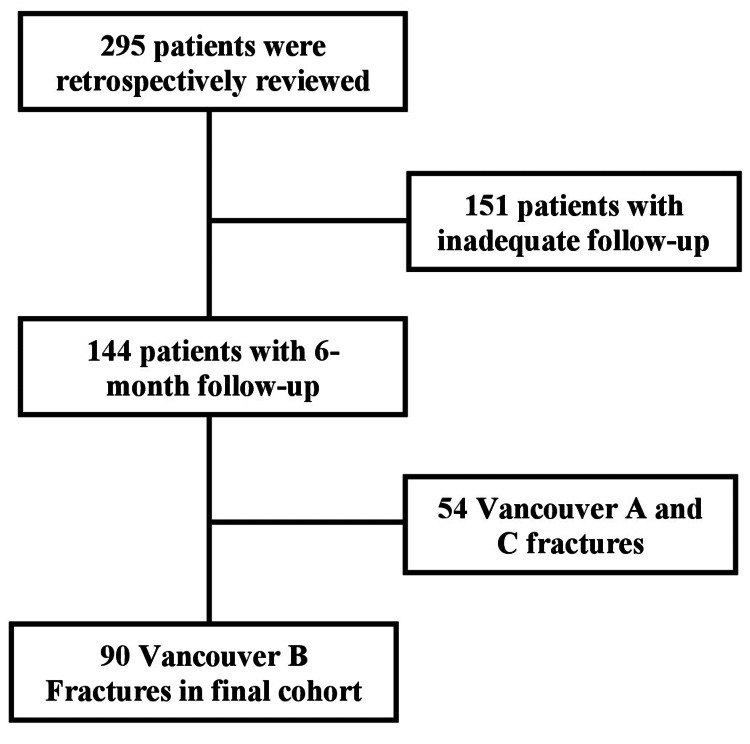
Flowchart of inclusion and exclusion criteria leading to final cohort

This retrospective multicenter chart review was conducted at two suburban level-I trauma centers. In total, 295 patients from 2007 to 2018 with periprosthetic femur fractures (with previous total hip arthroplasty or hemiarthroplasty) were reviewed. A patient list for review was created using ICD-9 code 996.44 (periprosthetic fracture around the prosthetic joint) and ICD-10 codes M97.01XA (periprosthetic fracture around the internal prosthetic right hip joint, initial) and M97.02XA (periprosthetic fracture around the internal prosthetic left hip joint, initial). Patients were included if they had Vancouver B periprosthetic femur fractures with previous hip arthroplasty, underwent primary surgical stabilization of their periprosthetic fracture, and had at least six months of postoperative follow-up. Patients were excluded if they sustained Vancouver A or C fractures (35 patients), had fractures that were treated nonoperatively (19 patients), underwent revision surgery for a repeat peri-implant fracture, or did not have adequate follow-up (151 patients) (Figure 1).

Overall, 90 patients met our inclusion criteria and were included in our cohort. The decision to utilize cerclage fixation as part of the construct was dependent on the surgeon and the fracture pattern. Patient demographics, injury details, fracture classification using the Vancouver system (by two fellowship-trained orthopedic trauma surgeons) [12-14], surgical details, fracture union, and postoperative complications were recorded for each patient. Of the 90 patients, 32 (35.6%) were B1, 42 (46.7%) were B2, and 16 (17.8%) were B3 fractures.

Data were cataloged using an electronic spreadsheet. Fracture union is defined as radiographic evidence of bridging callus seen across three out of four cortices on orthogonal femur films within six months of surgery. Delayed union was described as union achieved between six and nine months, and nonunion was described as those fractures that did not have radiographic or clinical union by nine months or underwent surgical intervention due to construct instability. Data between the two groups (fixation utilizing cerclage wiring and fixation without cerclage wiring) were compared using simple descriptive statistics and statistical significance tests (independent t-test, Fisher’s exact test, and chi-squared test) as appropriate to determine the superiority between revision techniques (IBM SPSS Statistics for Windows, Version 23, IBM Corp., Armonk, NY). For all analyses, p < 0.05 denotes statistical significance.

## Results

Overall, 90 patients were included in our study. A majority of our population was female (n = 59, 65.9%) and older than 71 years of age (mean = 74.5 years, median = 75.5 years) (n = 59, 65.6%) without a documented diagnosis of osteoporosis (n = 55, 61.1%), diabetes (n = 57, 63.3%), or smoking (n = 83, 92.2%) (Table 1). Most of these injuries occurred as a result of a low-energy (fall or injury sustained during ambulation) mechanism (n = 88, 97.8%) at a mean of 8.10 years after initial hip arthroplasty surgery. Before their injury, 68 (75.6%) patients had THA, and 22 (24.4%) had hemiarthroplasties, with 23 (25.6%) of these patients having cemented implants (Table 2).

**Table 1:** Demographic breakdown of the sample population No cerclage fixation includes those with fractures stabilized using revision arthroplasty, plate fixation, or a combination of the two, all without cerclage fixation.

		No Cerclage Fixation	Cerclage Fixation Only	Cerclage and Plate Fixation	Total
Gender	Male	11 (12.2%)	10 (11.1%)	10 (11.1%)	31 (34.4%)
	Female	11 (12.2%)	14 (15.6%)	34 (37.8%)	59 (65.6%)
Age	≤60 years	5 (5.6%)	4 (4.4%)	5 (5.6%)	14 (15.6%)
	61-70 years	7 (7.8%)	6 (6.7%)	4 (4.4%)	17 (18.9%)
	71-80 years	6 (6.7%)	10 (11.1%)	7 (7.8%)	23 (25.6%)
	≥81 years	4 (4.4%)	4 (4.4%)	28 (31.1%)	36 (40.0%)
Smoking	Yes	2 (2.2%)	2 (2.2%)	3 (3.3%)	7 (7.8%)
	No	20 (22.2%)	22 (24.4%)	41 (45.6%)	83 (92.2%)
Osteoporosis	Yes	20 (22.2%)	3 (3.3%)	12 (13.3%)	35 (28.9%)
	No	2 (2.2%)	21 (23.3%)	32 (35.6%)	55 (61.1%)
Diabetes	Yes	20 (22.2%)	6 (6.7%)	7 (7.8%)	33 (36.7%)
	No	2 (2.2%)	18 (20.0%)	37 (41.1%)	57 (63.3%)
Total		22 (24.4%)	24 (26.7%)	44 (48.9%)	90 (100.0%)

**Table 2:** Existing implants, fracture classification, and fixation construct of the sample population No cerclage fixation includes those with fractures stabilized using revision arthroplasty, plate fixation, or a combination of the two, all without cerclage fixation. THA: Total hip arthroplasty; Hemi: Hemiarthroplasty.

		No Cerclage Fixation	Cerclage Fixation Only	Cerclage and Plate Fixation	Total
Existing implant	THA	19 (21.1%)	20 (22.2%)	29 (32.2%)	68 (75.6%)
	Hemi	3 (3.3%)	4 (4.4%)	15 (16.7%)	22 (24.4%)
	Cemented	13 (14.4%)	2 (2.2%)	8 (8.9%)	23 (25.6%)
	Uncemented	9 (10.0%)	22 (24.4%)	36 (40.0%)	67 (74.4%)
Vancouver classification	B1	1 (1.1%)	0 (0.0%)	31 (34.4%)	32 (35.6%)
	B2	18 (20.0%)	20 (22.2%)	4 (4.4%)	42 (46.7%)
	B3	3 (3.3%)	4 (4.4%)	9 (10.0%)	16 (17.8%)
Total		22 (24.4%)	24 (26.7%)	44 (48.9%)	90 (100.0%)

Thirty-two (35.6%) of these fractures were classified as Vancouver B1, 42 (46.7%) as B2, and 16 (17.8%) as B3. Twenty-two patients (24.4%) were fixed without cerclage fixation (using revision arthroplasty, plate fixation, or a combination of the two), 24 (26.7%) with only cerclage fixation, and 44 (48.9%) with both cerclage wiring and plate fixation. Most patients being fixed with no cerclage wiring had both revision arthroplasty and plate fixation used (n = 15). Vancouver B1 fractures were mostly fixed with cerclage and plate fixation (31 of 32 patients) and retention of arthroplasty implants. Vancouver B2 fractures were mostly stabilized with no cerclage fixation (18 of 42 patients) or cerclage fixation only (20 of 42 patients) and revision arthroplasty, while Vancouver B3 fractures were mostly stabilized using cerclage and plate fixation (nine out of 16 patients) and revision arthroplasty (Table 2).

The average time to injury after the original implant was placed was 2,950 days (245.8 months), while the average time to surgery was 1.8 days. Though the time to injury was not statistically significant between the three groups (p = 0.26), the time to surgery was significantly different between no cerclage fixation (2.3 days), only cerclage fixation (1.1 days), and both cerclage and plate fixation (1.6 days) (p = 0.02). However, further bivariate logistic regression analysis did not identify the time to surgery as a significant correlator of union (p = 0.986). Furthermore, the average hospital length of stay postoperatively was 5.4 days and was also not significantly different between groups (p = 0.46).

**Figure 2:**
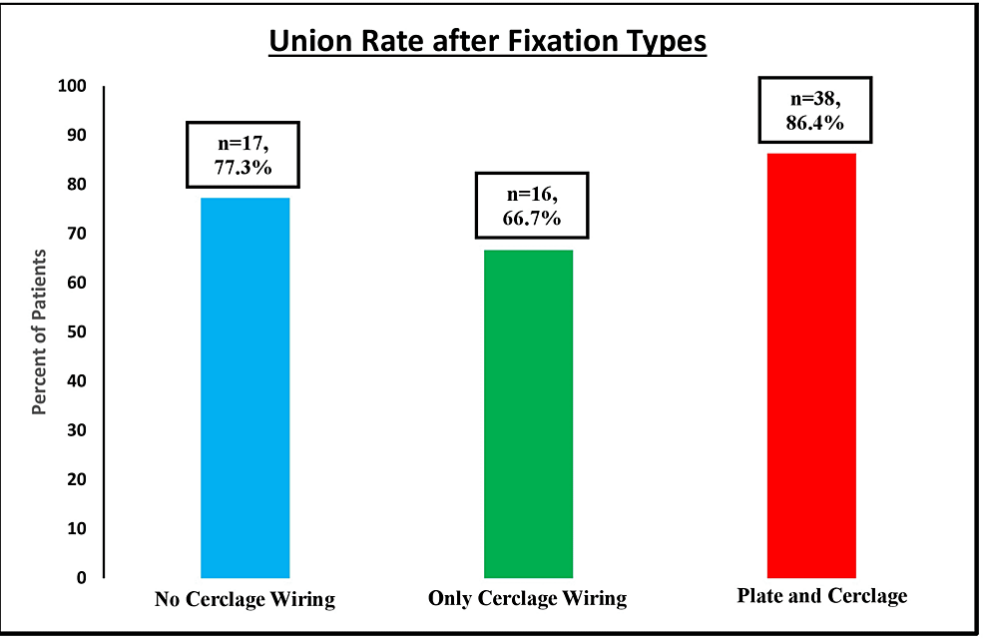
Union rate based on fixation types Statistical analysis was performed with chi-squared analysis. No statistically significant difference was seen in the union rates between the groups, whether this was analyzed using all three fixation groups (p = 0.38) or two fixation groups (p = 0.83).

Overall, 69 patients progressed to fracture union (76.7%), while two (2.2%) had a delayed union and 19 (21.1%) had a nonunion of their fracture (Figure 2). When comparing fractures stabilized without cerclage wiring versus all fractures stabilized with cerclage wiring (including only cerclage wiring and both cerclage wiring and plate fixation), there was no statistically significant difference in the union rates between the two groups (p = 0.83). This similarity held true when analyzing the union rate among the three groups of fixation types (no cerclage wiring, only cerclage wiring, and cerclage wiring and plate fixation) as well (p = 0.374). This analysis was done with patients divided into two groups, comparing patients who achieved union within six months and patients who did not. While statistical analysis could not be performed on B1 fractures due to the low sample size, there was no statistical significance between the union rates of fractures fixed including cerclage wiring and those without Vancouver B2 (42.9% versus 38.1%, p = 0.43) and B3 (37.5% versus 6.25%, p = 1.00) fractures (Table 3). This held true when comparing the union rates between the fractures fixed using only cerclage fixation (without plate fixation) and no cerclage wiring for B2 (p = 0.24) and B3 (p = 1.00) fractures as well.

**Table 3:** Comparison of union rates between surgical fixation that utilizes with cerclage and surgical fixation without cerclage Statistical analysis was performed using Fisher’s exact test. Fixation without cerclage includes revision arthroplasty alone or revision arthroplasty with plate osteosynthesis.

		Fixation Types		Total	p-value
		Fixation Including Cerclage	Fixation Without Cerclage		
B1	Union	27 (84.4%)	0 (0.0%)	32	N/A
	Nonunion	4 (12.5%)	1 (3.1%)		
B2	Union	18 (42.9%)	16 (38.1%)	42	0.43
	Nonunion	6 (14.3%)	2 (4.8%)		
B3	Union	6 (37.5%)	1 (6.25%)	16	1.00
	Nonunion	7 (43.8%)	2 (12.5%)		

**Figure 3:**
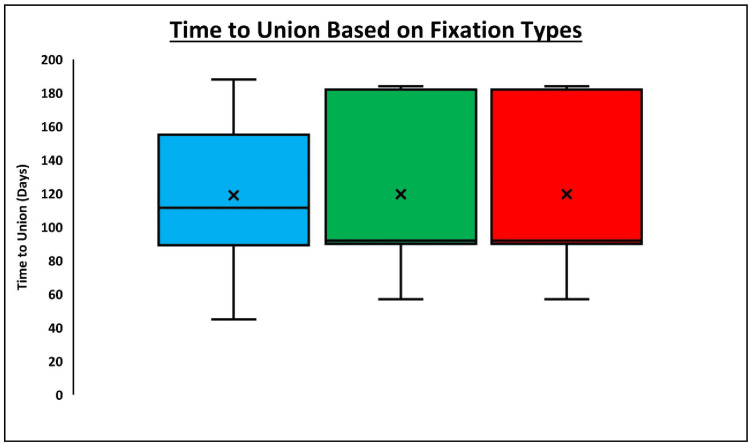
Time to union based on fixation types Statistical analysis was performed with the analysis of variance (ANOVA) test. There was no statistically significant difference seen in the time to union between the groups, whether this was analyzed using all three fixation groups (p = 0.91) or two fixation groups (p = 0.98).

Among the patients who achieved union, those who underwent fixation with no cerclage wiring achieved union at an average of 118.9 days. Patients who underwent fixation with only cerclage wiring achieved union at an average of 119.6 days, while those who underwent fixation with both cerclage and plate fixation achieved union at an average of 113.7 days (Figure 3). There was no statistically significant difference seen in the time to union between groups, whether this was analyzed using all three fixation groups (p = 0.91) or two fixation groups (p = 0.98). When stratifying by fracture type, there was no difference in the time to union among the three fixation (p = 0.84) or two fixation (p = 0.56) groups among Vancouver B2 fractures, while it was not possible to run this analysis among B1 and B3 fractures due to low sample size.

**Figure 4:**
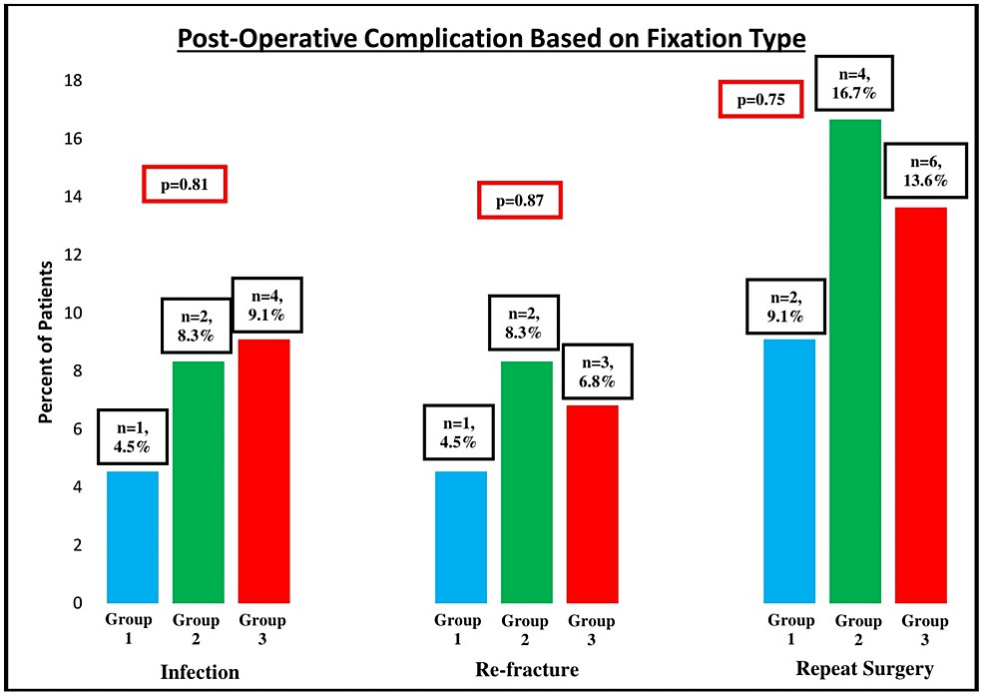
Postoperative complications based on fixation types Statistical analysis was performed with chi-squared analysis. Group 1 = No cerclage wiring; Group 2 = Only cerclage wiring; Group 3 = Cerclage wiring and plate fixation.

There was also no difference in three or two fixation group analysis in the infection rate (p = 0.81 and p = 0.51, respectively), re-fracture rate (p = 0.87 and p = 0.65, respectively), or re-operation rate (p = 0.75 and p = 0.50, respectively) (Figure 4). Finally, the bivariate analysis showed that fracture type (p = 0.14), history of osteoporosis (p = 0.38), history of diabetes (p = 0.52), current smoking status (p = 0.17), and implant type (p = 1.00) were not associated with significantly higher union rates. Age (p < 0.01) and gender (p = 0.01) were significantly correlated with union rates, with increasing age and female gender being associated with higher union rates (Table 4). Of note, smoking was not a significant predictor of union rates whether patients were divided into two groups (current smokers versus non-current smokers) or three groups (current smokers versus previous smokers who quit versus non-smokers).

**Table 4:** Risk factors for nonunion of periprosthetic femur fractures All values reported are absolute counts other than age, which is reported as a mean. Chi-squared test, Fisher’s exact test, and independent t-test were run as appropriate with p ≤ 0.05 denoting significance. THA: Total hip arthroplasty; Hemi: Hemiarthroplasty.

		Union	Delayed Union/Nonunion	P-value
Fracture type	B1	24	4	0.07
	B2	15	4	
	B3	5	5	
Osteoporosis	Yes	27	8	0.93
	No	42	13	
Diabetes	Yes	25	8	0.88
	No	44	13	
Current smoker	Yes	4	3	0.34
	No	65	18	
Gender	Male	19	12	0.01
	Female	50	9	
Implant type	THA	49	19	0.09
	Hemi	20	2	
Cemented	Yes	17	6	0.72
	No	52	15	
Age		76.8 years	67.1 years	<0.01

Finally, given the relatively low incidence of periprosthetic femur fractures, the authors did not run an a priori sample size analysis. A post hoc power analysis showed that our study was 4.2% powered. Assuming a minimal clinically important difference (MCID) of 5% in union rate as determined by our senior author, a properly powered study would need a cohort of over 1000 patients.

## Discussion

As our population grows older, periprosthetic femur fractures are becoming more common. As such, it is important to determine optimal fixation methods that safely and effectively stabilize the fracture and implant in hopes of improving the quality of life moving forward. Ample research has been performed on different fixation types based on the types of fracture [16-19]. Special consideration has been given to cerclage wiring [18-19] and its role in fracture fixation, especially after studies have shown that its utilization will not disrupt the periosteal blood supply from the femoral vessels [23]. This study analyzes clinical outcomes after cerclage wire fixation for Vancouver B fractures while investigating to see if there are any differences between this fixation method and other fixation methods not utilizing wire fixation. Biomechanical studies have theorized that plate fixation is a stronger construct than cerclage fixation alone [24], but the effect of these differences on clinical outcomes has not been explored.

Fractures in our cohort mostly occurred after low-energy injuries [7], the most common reported injury pattern, in elderly females [25]. Most of our patients also had THA that were uncemented [7], another known risk factor for periprosthetic fractures. These patients should be counseled about their higher risk for sustaining periprosthetic fractures in the future that could require further operative intervention. All Vancouver B1 fractures were treated with open reduction and internal fixation (plates and screws with or without cerclage fixation), while most B2 and B3 fractures were treated with a combination of revision arthroplasty and internal fixation [26]. However, six B2 and 3 B3 injuries were treated with internal fixation without revision arthroplasty, a published but less commonly used treatment option. Furthermore, three B2 and one B3 injuries underwent surgical stabilization with only revision arthroplasty [27], a less commonly utilized treatment option.

Our results indicate that fractures stabilized with cerclage fixation have similar clinical outcomes compared to those treated without cerclage wiring, confirming our initial hypothesis. This held true when comparing the entire cohort by fixation type as well as stratifying by Vancouver fracture type. This echoes the results that Moore et. al. noted in their studies exploring cerclage wire fixation in Vancouver B1 and C fractures [18]. Cerclage fixation or cables can be used around the bone only or more commonly around the plate and bone to help achieve stable fixation. In our cohort, stable fracture fixation was achieved with or without the use of cerclage wiring, resulting in similar union rates, time to union, re-fractures, and repeat operations among the two fixation groups. The authors believe that each fracture should be considered individually, with each having various satisfactory fixation options. Furthermore, there was no difference in infection rates, re-fracture rate, or repeat surgery rate between groups, even if there is a theoretical increased risk of infection with more implants placed. This further strengthens our hypothesis of clinical similarity between fixation types.

Finally, our study found that age and gender were the only demographic factors associated with lower rates of the union. Previous studies detail risk factors for fracture nonunion, including female gender, smoking, and cementless primary arthroplasty [28]. Some studies argue that increasing age and osteoporosis are risk factors for fracture nonunion [29,30]. While the significantly higher average age and female gender in our union group contradict these previous studies, these and all our results must be interpreted with caution due to the relatively low rate of fracture nonunion in our study. Our lack of correlation between smoking and union rates could be a product of the low number of current smokers in our cohorts. Furthermore, the rate of osteoporosis in our cohort could be underestimated due to incomplete charting or inadequate testing.

The strengths of this study include its multicenter nature involving two geographic areas and patient populations while expanding on the existing literature by evaluating all operative Vancouver B fractures. Vancouver A and C fractures were excluded because of their relatively lower incidence and need for operative fixation (with regard to Type A fractures).

The weaknesses of this study are inherent to its retrospective nature. Because of inadequate follow-up, our final sample is a low percentage of the total charts reviewed (30.5%). While the final sample size is small and inhomogeneous, it is similar to existing comparable studies [17,20]. Due to this fact, all patients were included rather than running an a priori power analysis. Bias introduced by surgeon operative technique based on preferences and training could not be accounted for. Finally, as with all retrospective chart reviews, data accuracy is dependent on accurate provider reporting.

## Conclusions

Periprosthetic femur fractures are common injuries, occurring after low-energy mechanisms in the elderly female population. While the Vancouver fracture pattern helps to guide the surgical construct used for fixation, the use of cerclage wires does not impact bony union in these injuries. Interestingly, increasing age and female gender were associated with increased union rates. Surgeons should individually consider each patient demographic as well as their fracture type when deciding which construct will achieve stable fixation that allows for fracture healing.
